# Contralaterally Controlled Functional Electrical Stimulation Combined With Brain Stimulation for Severe Upper Limb Hemiplegia—Study Protocol for a Randomized Controlled Trial

**DOI:** 10.3389/fneur.2022.869733

**Published:** 2022-04-29

**Authors:** Akhil Mohan, Jayme S. Knutson, David A. Cunningham, Morgan Widina, Kyle O'Laughlin, Tarun Arora, Xin Li, Ken Sakaie, Xiaofeng Wang, Ken Uchino, Ela B. Plow

**Affiliations:** ^1^Department of Biomedical Engineering, Lerner Research Institute, Cleveland Clinic, Cleveland, OH, United States; ^2^Department of Physical Medicine and Rehabilitation, MetroHealth System, Cleveland, OH, United States; ^3^Department of Physical Medicine and Rehabilitation, Case Western Reserve University, Cleveland, OH, United States; ^4^Louis Stokes Cleveland Veterans Affairs Medical Center, Cleveland FES Center, Cleveland, OH, United States; ^5^Krembil Research Institute, University Health Network, Toronto, ON, Canada; ^6^Department of Diagnostic Radiology, Imaging Institute, Cleveland Clinic, Cleveland, OH, United States; ^7^Respiratory Institute Biostatistics Core, Lerner Research Institute, Quantitative Health Sciences, Cleveland Clinic, Cleveland, OH, United States; ^8^Cerebrovascular Center, Neurological Institute, Cleveland Clinic, Cleveland, OH, United States; ^9^Department of Physical Medicine and Rehabilitation, Neurological Institute, Cleveland Clinic, Cleveland, OH, United States

**Keywords:** stroke, rehabilitation, motor function, brain stimulation, hemiplegia, transcranial magnetic stimulation, contralaterally controlled functional electrical stimulation, diffusion tensor image

## Abstract

**Background:**

Approximately two-thirds of stroke survivors experience chronic upper limb paresis, and of them, 50% experience severe paresis. Treatment options for severely impaired survivors are often limited. Rehabilitation involves intensively engaging the paretic upper limb, and disincentivizing use of the non-paretic upper limb, with the goal to increase excitability of the ipsilesional primary motor cortex (iM1) and suppress excitability of the undamaged (contralesional) motor cortices, presumed to have an inhibitory effect on iM1. Accordingly, brain stimulation approaches, such as repetitive transcranial magnetic stimulation (rTMS), are also given to excite iM1 and/or suppress contralesional motor cortices. But such approaches aimed at ultimately increasing iM1 excitability yield limited functional benefit in severely impaired survivors who lack sufficient ipsilesional substrate.

**Aim:**

Here, we test the premise that combining Contralaterally Controlled Functional Electrical Stimulation (CCFES), a rehabilitation technique that engages the non-paretic upper limb in delivery of neuromuscular electrical stimulation to the paretic upper limb, and a new rTMS approach that excites intact, contralesional higher motor cortices (cHMC), may have more favorable effect on paretic upper limb function in severely impaired survivors based on recruitment of spared, transcallosal and (alternate) ipsilateral substrate.

**Methods:**

In a prospective, double-blind, placebo-controlled RCT, 72 chronic stroke survivors with severe distal hand impairment receive CCFES plus cHMC rTMS, iM1 rTMS, or sham rTMS, 2X/wk for 12wks. Measures of upper limb motor impairment (Upper Extremity Fugl Meyer, UEFM), functional ability (Wolf Motor-Function Test, WMFT) and perceived disability are collected at 0, 6, 12 (end-of-treatment), 24, and 36 wks (follow-up). TMS is performed at 0, 12 (end-of-treatment), and 36 wks (follow-up) to evaluate inter-hemispheric and ipsilateral mechanisms. Influence of baseline severity is also characterized with imaging.

**Conclusions:**

Targeting of spared neural substrates and rehabilitation which engages the unimpaired limb in movement of the impaired limb may serve as a suitable combinatorial treatment option for severely impaired stroke survivors.

**ClinicalTrials No:**

NCT03870672.

## 1. Introduction

Approximately, 37–50% of stroke survivors live with chronic severe upper limb paresis which is characterized by limited active range of motion, strength, and coordination from the shoulder to the hand/fingers and severely diminished ability to perform activities of daily living (1). Many rehabilitation therapies, such as constraint-induced movement therapy (CIMT) or intensive task-oriented training, are not feasible for this sub-population due to the requirement of residual movement in wrist, thumb, and fingers. Therefore, developing rehabilitation interventions that are both effective and applicable for stroke survivors with severe upper limb motor impairment is a major unmet clinical need.

Contralaterally controlled functional electrical stimulation (CCFES) is a therapy that can be used in stroke survivors with little to no residual hand/finger extension (2). CCFES delivers neuromuscular electrical stimulation to finger and thumb extensors to open the paretic hand with a stimulation intensity that is proportional to the degree of volitional opening of the non-paretic hand wearing an instrumented glove. Therefore, as patients open their non-paretic hand, the paretic hand opens as well to allow participation in task-oriented practice. In a recent RCT including chronic stroke survivors with moderate and severe upper limb impairment (*N* = 80), CCFES led to greater improvements in dexterity compared to cyclic neuromuscular electrical stimulation (cNMES) at 6-months post-treatment. Though significant between-group differences in upper limb motor impairment (Upper Extremity Fugl-Meyer; UEFM) were not seen, unpublished secondary analyses reveal UEFM gains were in favor of the CCFES group in severely impaired participants (*N* = 28). Participants with severe impairment at baseline experienced greater UEFM gains at 6-weeks after CCFES, 2.8 (95% CI, 0.8 to 4.8) than after cNMES, 0.8 (95% CI, −0.3 to 1.9), *p* = 0.09. UEFM gains were even larger in patients with severe impairment who were < 2years post-stroke, 4.5 (95% CI, 2.4 to 6.6) after CCFES vs. 0.8 (95% CI, −0.6 to 2.3) after cNMES, *p* = 0.006. Based on minimal clinically important difference (MCID) of 4.25 for UEFM (3), 50% of severely impaired patients < 2years post-stroke, who had received CCFES, attained clinically meaningful UEFM gains, compared to 9% of participants, who had received cNMES, *p* = 0.06. But since this advantage seen on UEFM after CCFES did not translate to improvement in functional abilities (Arm Motor Ability Test) across severely impaired stroke survivors, an opportunity exists to build upon the gains achieved by augmenting CCFES with another technique that may enhance the neuroplastic effects of CCFES.

Noninvasive repetitive transcranial magnetic stimulation (rTMS) can enhance functional gains associated with rehabilitation. Cortical areas involved in plastic mechanisms are targeted to potentiate their contribution toward recovery (4, 5). High-frequency rTMS (≥5*Hz*) can increase cortical excitability, while low-frequency rTMS (≤ 1*Hz*) can suppress cortical excitability. In stroke, ipsilesional primary motor cortex (iM1) is the most common target. Typically, high-frequency rTMS is delivered to increase excitability of iM1 directly, or low-frequency rTMS is given to suppress the inhibitory influence of contralesional motor areas and thereby increase excitability of iM1 indirectly. Regardless, severe damage to ipsilesional corticospinal pathways limit gains in function that can be made with iM1 facilitation (6, 7).

Our group and subsequently other groups have tested a new approach to promote motor function in survivors with more severe motor impairment and injury. This approach involves facilitating the excitability of contralesional higher-motor cortices (cHMC) with high-frequency rTMS, based on the view that these regions can make positive contributions to ipsilateral (paretic) upper limb movement *via* extensive bi-hemispheric connections and alternate, uncrossed pathways in the absence of sufficient ipsilesional substrate (8–10). Transient disruption of cHMC activity produces greater impairment in movement of the paretic limb in stroke survivors with more severe motor impairment (10–12). Transient disruption of iM1 activity does not have such an effect, indicating cHMC may be crucial for behavioral restitution in survivors with severe motor impairment and injury. Likewise, we have found that facilitation of cHMC activity with high-frequency rTMS produces greater improvement in motor control than facilitation of iM1 in survivors with more severe motor impairment, reinforcing the value of these intact targets in recovery (11, 13).

Therefore, we hypothesized that CCFES when combined with facilitation of cHMC *via* high-frequency rTMS would produce synergistic gains in motor function in survivors with more severe motor impairment. CHMC would be a favorable target not just based on its potential to make positive contributions to paretic limb movement in the absence of sufficient ipsilesional substrate but also because of its specialized role in bilateral control, which underlie CCFES-mediated training (8, 10).

In an ongoing randomized controlled trial of stroke survivors with severe chronic hand motor impairment, we are testing whether CCFES combined with rTMS facilitation of cHMC produces a greater reduction in motor impairment and perceived disability, and larger gains in functional ability as compared to CCFES combined with rTMS facilitation of iM1 and CCFES combined with sham rTMS (**Hyp 1**). CCFES with rTMS facilitation of cHMC will produce greater motor functional gains achieved with increases in the excitability of uncrossed pathways and bi-hemispheric connections (**Hyp 2**). We anticipate the severity of baseline impairment and extent of corticospinal damage will mediate the overall treatment effects (**Hyp 3**).

## 2. Methods

### 2.1. Participants

This study is a randomized, sham-controlled, and double-blinded clinical trial of up to 72 participants who had suffered a stroke at least 6 months previously and who continue to experience severe chronic upper limb motor impairment. Clinical assessment of the motor impairment is performed by an occupational therapist. “Severe” motor deficit is being defined as the inability to extend wrist ≥10-degrees, extend/abduct thumb ≥10-degrees, or extend two other digits ≥10-degrees (exclusion criteria in CIMT studies). This clinical motor criterion was chosen because it has a better prospect of being generalizable and was also used to define “severe” motor impairment in the previous CCFES clinical trial (2). Participants are required to have functional paretic hand opening in response to neuromuscular electrical stimulation of finger or thumb extensors to ensure it is feasible for them to undergo CCFES-mediated rehabilitation (See Inclusion Criteria, Table 1). Participants are excluded for contraindications to TMS and magnetic resonance imaging (MRI), including cardiac pacemaker, history of seizures, substance abuse, metallic implant in the head etc. determined by a stroke neurologist (See Exclusion Criteria, Table 2). Participants are being recruited from the Cleveland Clinic, Cleveland, OH. The trial is governed by human research protection policies of the Cleveland Clinic Institutional Review Board. All participants provide written informed consent before participating.

**Table 1:** Inclusion criteria.

• Age 18–90 years old at the time of randomization
• >6 months since a first clinical cortical or subcortical, ischemic or hemorrhagic stroke
• Severe upper limb hemiparesis defined as lacking ≥10-degrees active wrist extension or ≥10-degrees active thumb abduction/extension or ≥10-degrees active extension in at least two additional digits (i.e., will not meet minimum constraint induced movement therapy criteria)
• Memory of at least 2 out of 3 items after 30 min
• Able to perform 3-stage command
• Full volitional hand opening/closing of the non-paretic hand
• Ability to follow instructions for putting on and operating the contralaterally controlled functional electrical stimulation (CCFES) device or have a caregiver available to provide assistance
• Adequate active movement of shoulder and elbow to position the paretic hand on one's lap for performance of functional task practice and CCFES-assisted hand opening exercises
• Skin intact on hemiparetic arm
• Surface electrical stimulation of the paretic finger and thumb extensors produces functional hand opening without pain (this will exclude patients who have too much flexor spasticity)
• Able to hear and respond to cues from stimulator
• Completed occupational therapy at least 2 months prior to enrollment (no concomitant OT)

**Table 2:** Exclusion criteria.

• Concomitant neurologic diagnosis of peripheral nerve injury, Parkinson's disease, spinal cord injury, traumatic brain injury, or multiple sclerosis
• Brainstem stroke
• Severe shoulder or hand pain, i.e., unable to position hand in the workspace without pain
• Insensate to touch on forearm or hand
• Uncompensated hemi-neglect (extinguishing to double simultaneous stimulation)
• Cardiac pacemaker or other implanted electronic system
• History of potentially fatal cardiac arrhythmia
• Diagnosis (apart from stroke) that substantially affects paretic arm and hand function
• Deficits in communication that interfere with reasonable study participation
• Lacking sufficient visual acuity to see the stimulator's display
• Concurrent enrollment in another investigational study
• Pregnancy
• Metal implants in the head
• Seizure as an adult or diagnosed with epilepsy
• Current abuse of alcohol or illicit drugs
• Botulinum toxin injections to any upper extremity muscles within 2 months of enrolling
• Taking any anti-convulsant
• Taking bupropion/valbutrin (other antidepressants are acceptable)
• History of fainting spells of unknown/undetermined etiology
• Implanted pumps, deep brain stimulator, shunts, nerve stimulators
• Current diagnosis of carpal tunnel syndrome
• Previous adverse reaction to transcranial magnetic stimulation

### 2.2. Randomization

Participants are assigned to one of three treatment groups—CCFES plus 5 Hz rTMS of cHMC, CCFES plus 5 Hz rTMS of iM1, or CCFES plus sham rTMS of cHMC/iM1 using an adaptive randomization algorithm (14, 15). The algorithm is designed to minimize group imbalances on 4 key participant characteristics: (1) time post-stroke (<2 years vs. ≥2 years), (2) presence of cortical vs. subcortical lesion, (3) presence of active wrist extension vs. none and (4) paresis of dominant vs. non-dominant side. Two years post-stroke was chosen as stratification cutoff because our previous CCFES trial had reported a higher likelihood of response in participants within that time-frame (2). With the inclusion of the wrist extension, we expect that baseline differences in degree of distal upper limb severity will be minimized. Balancing across the three groups can still be challenging. Allocation is concealed by storing group assignments in a binder to which only the study coordinator and rTMS interventionists have access.

### 2.3. Blinding

The assessors, the therapist, and the participants are all blinded to group assignment. Though participants are aware of which hemisphere the rTMS is being applied, they are not aware of whether real or sham rTMS is being delivered. Stimulation parameters (intensity, frequency and the total number of pulses), stimulation site (iM1 or cHMC) and type of stimulation (real or sham) are verified by rTMS interventionists before rTMS is delivered. Integrity of blinding is determined at the end of the intervention period using a questionnaire which requires participants and assessors to guess the group allocation.

## 3. Intervention

For all groups, treatment lasts 12 weeks and consists of 22 sessions of group-specific rTMS for 21 min followed immediately by 1 h of therapist-guided CCFES-mediated functional task practice performed in the laboratory (two sessions per week except on weeks 6 and 12, which include an assessment session). In addition to this, participants self-administer 10 sessions per week of CCFES-mediated repetitive hand opening exercises at home (no more than two sessions per day separated by at least 2 h).

### 3.1. rTMS Therapy

RTMS is delivered according to the group assignment using MagStim^®^ *Rapid*^2^ device (The Magstim Company Ltd, UK) with the D70 AirFilm figure-of-eight coil (AFC, S/N0738). Bi-phasic rTMS pulses are delivered with a pulse frequency of 5 Hz to facilitate the excitability of cHMC or iM1. Forty-two, 10-s trains of 50 pulses each are delivered at an inter-train interval of 20.3 s for a total of 2,100 pulses. Sham rTMS is delivered using another D70 AirFilm figure-of-eight sham coil (AFC Sham, S/N0101). Real rTMS elicited the activation of the targeted cortical region, while sham rTMS did not have any such physiological effect; however, the acoustic artifacts are present during real and sham stimulation. rTMS targeting is guided by MRI-stereotaxy.

Prior to starting rTMS, surface 1.8 inch diameter EMG electrodes (Adult Tape ECG Ultratrace^®^ Wet Gel, Conmed Corporation, USA) are placed over the extensor digitorum communis (EDC) and biceps brachii (BB) muscles of the side contralateral to rTMS site. TMS “motor hotspot” for EDC is identified as the location at which the lowest TMS intensity generates motor evoked potentials (MEPs) ≥100μ*V* above slight baseline contraction (20–30% of maximum voluntary contraction) in 6 of 10 trials—also termed as active motor threshold (AMT). AMT is expressed as %maximum stimulator output (%MSO). rTMS is subsequently delivered at a sub-threshold intensity of 90%AMT. If ipsilesional AMT cannot be defined due to the absence of MEPs, rTMS is applied at 72% MSO, a common and safe practice in the study of stroke survivors with severe impairment (11).

In the iM1 group, rTMS is delivered to the ipsilesional motor hotspot, unless that hotspot cannot be identified, in which case mirror location of the contralesional motor hotspot is targeted. In the cHMC group, rTMS is targeted to a region 2 cm anterior and 1 cm medial to the contralesional motor hotspot, corresponding to the location of premotor cortex (PMC) and adjoining supplementary motor area (SMA) complex (16). In the sham group, rTMS is targeted to the iM1 site or the cHMC site (randomized assignment).

### 3.2. CCFES Therapy

Immediately after completing rTMS, participants engage in 1 h of therapist-guided, CCFES-mediated functional task practice in the laboratory. The CCFES stimulator delivers biphasic rectangular current pulses at a pulse frequency of 35 Hz and pulse amplitude of 40 or 60 mA (2). A pulse frequency of 35 Hz was chosen because it creates fused muscle contractions without inducing muscle fatigue as rapidly as higher frequencies (17). Up to 3 channels of stimulation are programmed to produce hand opening. Surface electrodes are positioned to activate the EDC and extensor pollicis longus (EPL). Abductor pollicis brevis (AbPB), dorsal interossei (DI), or extensor indicis proprius (EIP) may also be targeted if they are necessary to achieve functional hand opening. This process of determining electrode locations and stimulus parameters is done only once, and in a separate session prior to the first rTMS and CCFES therapy session. The CCFES stimulator is programmed to increase pulse duration for each stimulating electrode in proportion to the amount of opening of an instrumented glove worn on the contralateral non-paretic hand.

Early sessions of CCFES therapy focus on simpler tasks, such as practicing opening the hand adequately to acquire an object. Task difficulty progresses from easy-to-acquire-and-manipulate to tasks requiring wider hand opening, greater complexity, skill, and coordination of hand function with proximal upper limb movement. Hands-on assistance and strengthening are provided as necessary to reinforce correct movement patterns. Weight bearing and stretching are used to overcome spasticity and allow movements that are more effective.

### 3.3. Self-Administered Home Therapy

An instruction manual is provided to every participant, and they are trained to perform CCFES hand opening exercises at home. Each home session consists of three 15-min periods separated by 3 min of rest (45 min of work per session). During each 15-min period, the stimulator issues audio and visual cues prompting the participant to attempt to fully open both hands for several seconds, then relax both hands for several seconds, and repeat the cycle. Adherence to the home stimulation regimen is monitored with participant diaries and electronic data logging by the stimulator at every visit during the treatment period. The therapist checks patient diaries and logs stimulator usage data before beginning each therapy visit in the laboratory. In addition, the therapist monitors how patient engages in CCFES-based exercises at home by observing them in the laboratory while performing CCFES ( 10 min). The mean (SD) percentage of compliance of CCFES device use was recorded to be 96% (8.7%) (2) in our previous study and we expect similar results in our ongoing study.

## 4. Outcome Measures

**Figure 1:**
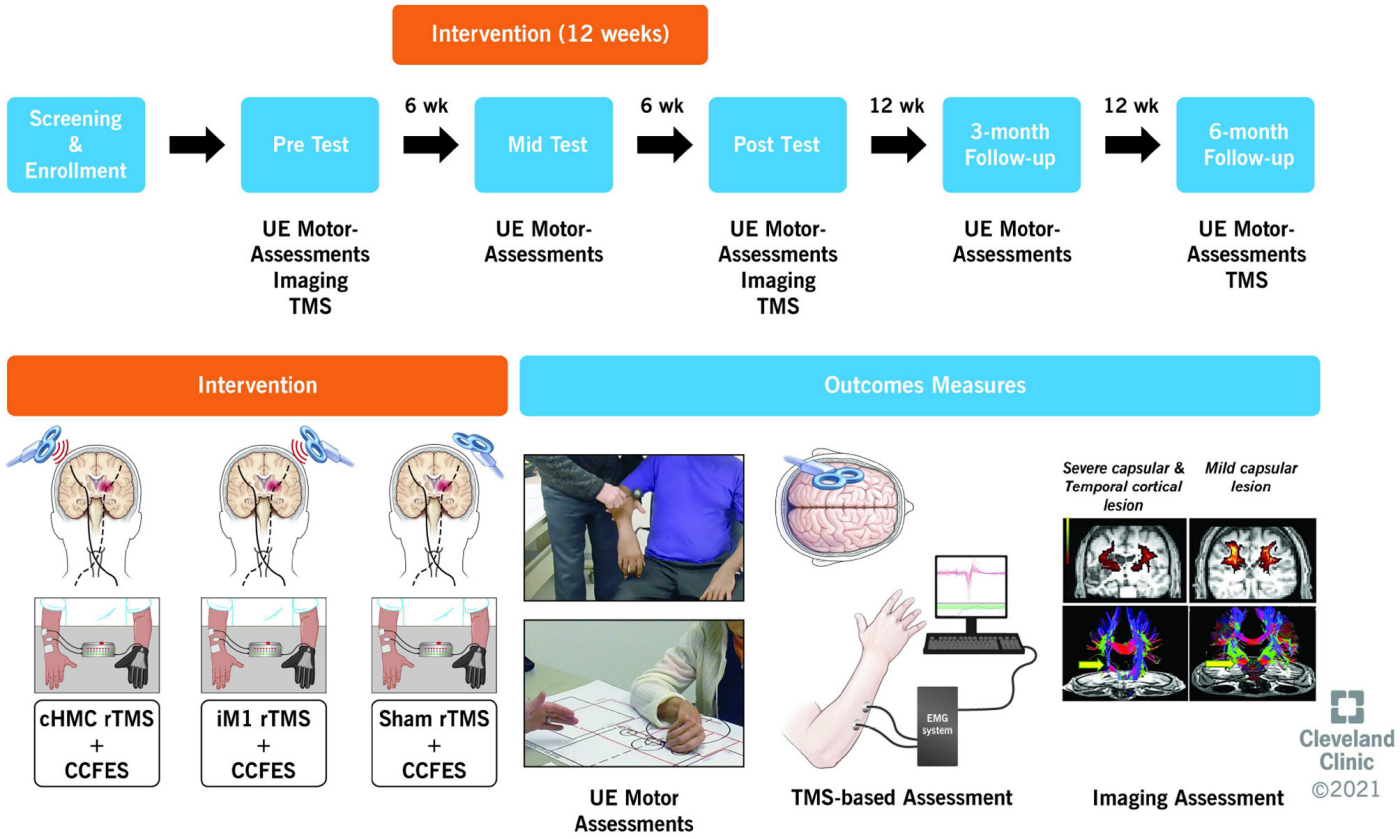
Overview of study design showing the timeline, intervention, and outcome measures. UE, upper extremity; TMS, transcranial magnetic stimulation; CCFES, contralaterally controlled functional electric stimulation.

Upper limb impairment, activity limitation, and patient-reported disability are assessed at 0 (baseline), 6 (mid-treatment), 12 (end-of-treatment), 24 (3-month follow-up), and 36 weeks (6-month follow-up) (Figure 1). Neurophysiological assessments are performed at 0, 12, and 36 weeks, on a separate day from the motor assessments. Imaging assessments are performed at 0 and 12 weeks (See Schedule of Assessments, Table 3). Subjects refrain from using their CCFES stimulators for 24 h prior to their 6-week and 12-week assessments in order to avoid any transient carry over effects or muscle fatigue.

**Table 3:** Schedule of assessments.

**Outcome measures**	**Week**				
	**0**	**6**	**12**	**24**	**36**
**Motor Impairment and Function Assessments**					
Upper Extremity Fugl Meyer (UEFM)	X	X	X	X	X
Wolf Motor Functional Test (WMFT)	X	X	X	X	X
Stroke Upper Limb Capacity Scale (SULCS)	X	X	X	X	X
Stroke Impact Scale (SIS-16)	X	X	X	X	X
Range of Motion (ROM)	X	X	X	X	X
Modified Ashworth Scale (MAS)	X	X	X	X	X
Patient Health Questionnaire (PHQ-9)	X	X	X	X	X
Leisure-Time Exercise Questionnaire (LTEQ)	X	X	X	X	X
**Neurophysiological and Motor Control Assessments**					
Transcranial Magnetic Stimulation (TMS)	X		X		X
Hoffman-Reflex (H-Reflex)	X		X		
Grip Force	X		X		X
Maximum Voluntary Contraction (MVC)	X		X		X
Bilateral Task	X		X		
**Neuroimaging Assessments**					
Magnetic Resonance Imaging (MRI)	X		X		
Diffusion Tensor Imaging (DTI)	X		X		
Functional MRI (fMRI)	X		X		
Resting state fMRI (rs-fMRI)	X		X		
**Self-Assessment of Treatment**					
Blinding Integrity			X		
End of Treatment Questionnaire			X		

*X indicates that the outcome measure will be assessed at that time point*.

### 4.1. Primary Outcomes

The primary endpoint is the change in upper-limb motor impairment at the end-of-treatment, as assessed by the Upper Extremity Fugl Meyer (UEFM) score. The UEFM is a reliable and valid measure of post-stroke upper limb motor impairment (18) that evaluates a participant's ability to perform upper extremity motions both in and out of synergy and with respect to both proximal and distal joints. The minimal clinically important difference (MCID) for UEFM in chronic stroke survivors with minimal to moderate impairment is a 4.25-point gain (3) and achievement of this at end-of-treatment will qualify as a clinically significant improvement/response. Other definitions of therapeutic response will also be compared to find convergence, including attaining response at secondary endpoints (12-wk, 24-wk follow-up) or a composite score based on impairment and functional ability measures or treatment induced gain.

### 4.2. Secondary Outcomes

#### 4.2.1. Behavioral Outcome Measures

Secondary measures listed in priority order include Wolf Motor Function Test (WMFT) rate for measuring motor function (19), Stroke Upper Limb Capacity Scale (SULCS) for measurement of upper-limb capacity (20), Modified Ashworth Scale (MAS) for evaluation of spasticity (21), grip strength (dynamometry), range of wrist extension, Stroke Impact Scale (SIS-16) for measuring patient-reported disability (22), and Leisure-time Exercise Questionnaire for measuring physical activity (23).

### 4.3. Neurophysiological Assessments

Neurophysiologic assessments are performed using TMS (Magstim 200^2^, The Magstim Company, U.K.). A 70 mm figure-of-eight TMS coil capable of delivering 100μs monophasic pulses is positioned tangentially over the scalp at locations corresponding to motor hotspots while participants are seated with their forearms supported on a flat surface. Coil placement and targeting are guided using MRI-stereotaxy. Surface 1.8 inch diameter EMG electrodes (Adult Tape ECG Ultratrace^®^ Wet Gel, Conmed Corporation, USA) are applied over the paretic and non-paretic EDCs to record MEPs and changes in ongoing muscle activity. EDC is the muscle of choice because it is the primary target muscle for CCFES, and return of MEPs in EDC will signify restoration of substrates mediating finger extension, a movement generally impaired after stroke and one that carries high clinical value (24). TMS is used to assess inter-hemispheric inhibition (IHI) and excitability of ipsilateral (uncrossed) pathways to the paretic hand. IHI is measured using the ipsilateral silent period (iSP) method (25). iSP is believed to reflect IHI imposed from the targeted motor cortex upon activity of the active ipsilateral limb (25). Excitability of ipsilateral (uncrossed) pathways to paretic muscles are measured using ipsilateral MEPs (iMEPs) evoked with TMS pulses delivered to contralesional motor regions. iMEPs are collected from the paretic biceps brachii (BB) muscle (in addition to the paretic EDC muscle) because uncrossed pathways preferentially innervate motoneurons devoted to proximal flexor muscle groups (26). Additional secondary assessments include metrics of excitability of ipsilesional and contralesional pathways to paretic and non-paretic EDC muscles, respectively (See Schedule of Assessments, Table 3).

### 4.4. Imaging Assessments

Imaging is performed on a Siemens Prisma 3T MRI with a standard 20 channel head-neck array coil (Siemens Healthineers, Erlangen, Germany). T1 weighted MPRAGE images at 1 mm isotropic resolution are acquired to determine lesion location and volume. Lesion location is categorized as involving the posterior limb of the internal capsule (PLIC), other subcortical structures, and/or cortices.

Diffusion tensor imaging (DTI) is also performed to assess white matter integrity of corticospinal tracts (27). A high angular resolution diffusion imaging (HARDI) sequence is acquired at 2 mm isotropic resolution. A unit-less measure of tissue integrity, Fractional anisotropy (FA) is measured at the level PLIC because it represents a major convergence point for corticospinal tracts (28) and is predictive of motor impairment, and functional gains (29).

## 5. Data and Safety Monitoring

A separate Trial Operations Committee monitors ethical and regulatory aspects, scientific integrity and fiscal integrity of the trial. An independent medical monitor (stroke neurologist) is assigned to the trial. The monitor reviews the study data, evaluates treatments for adverse events, judges whether the overall integrity and conduct of the study remain acceptable, and makes recommendations to the Trial Operations Committee. A quarterly review is performed to update the committee on progress of the trial, review enrollment, dropouts, data collection, and safety (adverse events). All adverse events are reported to the local IRB and categorized as serious/not serious, related/not related, and expected/unexpected. All staff associated with the study are required to complete human subject research training through the Cleveland Clinic and similar NIH programs.

## 6. Sample Size Determination

The estimated CCFES + Sham rTMS vs. CCFES + cHMC facilitation effect size is 0.87, calculated from common standard deviation from our previous studies, 6 (2), with expectation of a between-group difference of UEFM, 5.2 [minimal clinically important difference, MCID (3)]. With this effect size, a type I error of 0.05 and power of 80%, 22 subjects are needed per group. With an expected attrition rate of 10%, 72 is the planned sample size to ensure 22 per group to complete the study.

## 7. Statistical Analysis

All statistical analyses will be performed using the intent-to-treat principle, comparing outcomes by the assigned groups (linear mixed model analysis). The primary endpoint will be change in UEFM score from baseline to end-of-treatment at 12 weeks. Changes in UEFM, WMFT-rate, SIS-16 and neurophysiologic metrics will be modeled using a linear mixed effects approach, which is well-suited for handling correlated repeated measurements, unbalanced data, missing data and dropouts and also permits us to control for potential confounders (30). As secondary analysis, “responders” will be identified among the three groups, with response defined as the attainment of ≥4.25 point-gain on the UEFM (3). As secondary analysis, “responders” will be identified among the three groups, with response defined as the attainment of ≥4.25 point-gain on the UEFM (3).

The adjusted least square mean estimates from the linear mixed effects models will be used to compare the effectiveness of the CCFES + cHMC rTMS to CCFES + iM1 rTMS, and CCFES + sham (**Hyp 1**). Linear regression models will be used to evaluate the association between changes in neurophysiologic and motor outcomes (**Hyp 2**). Separate regression models will be used to determine if treatment response is associated with baseline impairment, lesion size/location and corticospinal damage (**Hyp 3**).

## 8. Discussion

Our ongoing clinical trial represents the first to investigate the effects of multiple sessions (22 sessions over 12 weeks) of CCFES-mediated therapy combined with rTMS facilitation of the contralesional higher motor cortices (cHMC) in chronic stroke survivors with severe hand impairment. Up until now, the effects of this novel approach have been only evaluated in single sessions (11, 13). The anticipation is that multiple sessions over several weeks would be beneficial for return of motor function in a population that requires longer and more intense training for motor improvement.

CHMC make positive contributions to paretic limb movement in the absence of sufficient ipsilesional pathways and is also specialized for bilateral movements (8–10). So, we hypothesize that combining CCFES-based therapy, which involves simultaneous opening and closing of both hands, with facilitation of cHMC will produce greater gains in motor recovery in stroke survivors with severe hand impairment than combining CCFES with facilitation of iM1 or sham brain stimulation. Superior effects of combining CCFES with cHMC-facilitation would validate our hypothesis that cHMC is a suitable target to promote motor function in the more severely impaired population (**Hyp 1**). If gains in motor function are achieved with CCFES + facilitation of cHMC and these gains are associated with increases in excitability of uncrossed pathways and rebalanced IHI, this would provide evidence of neuroplastic mechanisms specific to behavioral restitution for persons with more severe motor impairment (**Hyp 2**). Identifying which recovery phenotypes are associated with a favorable response (**Hyp 3**) would help identify biomarkers to select the best candidates for future larger efficacy trials.

Identifying the value of any approach that is specific to promoting motor function in stroke survivors with severe neurological damage and motor impairment potentially has far-reaching impact. After all, these individuals typically have limited opportunities for significant recovery, and treatments commonly given in clinics or tested in research are not feasible to make available to them (generally because criterion level of movement required is lacking). By including those stroke survivors who otherwise have too severe of a hand impairment to qualify for evidence-based therapies like CIMT, we seek to generalize the use of a potentially promising approach like brain stimulation. Instead of using a brain stimulation approach that is contingent upon the presence of residual ipsilesional pathways (iM1 facilitation), we propose here the use of a novel approach that relies on targeting what is intact in persons with severe (unilateral) ipsilesional damage. In this way, we seek to capitalize on “what remains” and not “what once was”, a critical step in developing targeted treatments.

Several safeguards are included to ensure the scientific rigor, reproducibility, and transparency of our findings. These include: inclusion of a homogeneous sample of more severe participants based on a clinical criterion (lack of minimal extension at fingers/wrist) for improving the statistical power of detecting between-group differences; application of CCFES in all groups with one of the groups receiving sham rTMS to differentiate the gains achieved by CCFES alone; multiple, assessor-blinded assessments performed before, during and after the end of the intervention to capture trends of motor improvement and retention of those effects for 6 months; and inclusion of baseline lesion characteristics, demographic and neurologic data, and treatment adherence in analytic models to improve variance in treatment effects.

## 9. Summary and Conclusions

We hypothesize that the combination of CCFES with rTMS facilitation of cHMC will produce greater reduction in motor impairment and perceived disability, and larger gains in functional ability compared to CCFES with rTMS facilitation of iM1 and CCFES with sham rTMS. Findings in line with our hypothesis would reveal that intact contralesional regions make more significant contributions to movement recovery than damaged ipsilesional regions in chronic stroke survivors with severe damage and impairment.

## Ethics Statement

The studies involving human participants were reviewed and approved by Cleveland Clinic Institutional Review Board. The patients/participants provided their written informed consent to participate in this study.

## Author Contributions

EP, JK, DC, KS, XW, and KU conceived the study. JK, DC, KS, KU, MW, KO'L, TA, XL, AM, and EP were involved in protocol development and implementation. AM, MW, XL, TA, and KO'L were involved in data collection. AM and XL were involved in data analysis. AM wrote the first draft of the manuscript and EP revised it. All authors reviewed and edited the manuscript and approved the final version of the manuscript.

## Funding

The work was supported by the National Institutes of Health and National Institute of Child Health and Human Development (NICHD) (Grant Number R01HD098073).

## Conflict of Interest

JK is an inventor on the CCFES patent assigned to Case Western Reserve University, Patent 8,165,685: System and Method for Therapeutic Neuromuscular Electrical Stimulation. This patent was licensed to Synapse Biomedical Inc. (Oberlin, Ohio) on February 1, 2019. The remaining authors declare that the research was conducted in the absence of any commercial or financial relationships that could be construed as a potential conflict of interest.

## Publisher's Note

All claims expressed in this article are solely those of the authors and do not necessarily represent those of their affiliated organizations, or those of the publisher, the editors and the reviewers. Any product that may be evaluated in this article, or claim that may be made by its manufacturer, is not guaranteed or endorsed by the publisher.
